# 3-(4-Meth­oxy­phen­yl)-1-phenyl-1*H*-pyrazole-4-carbaldehyde

**DOI:** 10.1107/S1600536811041808

**Published:** 2011-10-12

**Authors:** R. Prasath, P. Bhavana, Seik Weng Ng, Edward R. T. Tiekink

**Affiliations:** a Department of Chemistry, BITS, Pilani – K. K. Birla Goa Campus, Goa 403 726, India; b Department of Chemistry, University of Malaya, 50603 Kuala Lumpur, Malaysia; c Chemistry Department, Faculty of Science, King Abdulaziz University, PO Box 80203 Jeddah, Saudi Arabia

## Abstract

Four independent mol­ecules comprise the asymmetric unit of the title compound, C_17_H_14_N_2_O_2_. The central pyrazoline ring is flanked by an N-bound benzene ring and a C-bound meth­oxy-substituted benzene ring. The greatest difference between the independent mol­ecules is found in the relative orientations of the benzene rings with the range of dihedral angles being 23.59 (6)–42.55 (6)°. In the crystal, extensive C—H⋯O inter­actions link mol­ecules into layers parallel to (02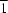) and these are linked by C—H⋯π contacts.

## Related literature

For background details and the biological applications of pyrazolines, see: Ali *et al.* (2007 ▶); Kaushik *et al.* (2010 ▶); Krishnamurthy *et al.* (2004 ▶). For a related structure, see: Prasath *et al.* (2011 ▶).
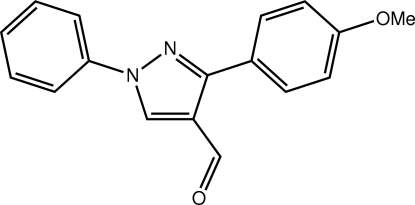


## Experimental

### 

#### Crystal data

C_17_H_14_N_2_O_2_
*M* *_r_* = 278.30
Triclinic, 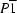

*a* = 9.9002 (3) Å
*b* = 17.1297 (4) Å
*c* = 17.1976 (4) Å
α = 80.842 (2)°
β = 89.373 (2)°
γ = 73.216 (2)°
*V* = 2754.77 (12) Å^3^
*Z* = 8
Cu *K*α radiation
μ = 0.72 mm^−1^
*T* = 100 K
0.22 × 0.20 × 0.08 mm

#### Data collection

Agilent SuperNova Dual diffractometer with an Atlas detector
Absorption correction: multi-scan (*CrysAlis PRO*; Agilent, 2010 ▶) *T*_min_ = 0.857, *T*_max_ = 0.944
37501 measured reflections
11521 independent reflections
10089 reflections with *I* > 2σ(*I*)
*R*_int_ = 0.040

#### Refinement

*R*[*F* ^2^ > 2σ(*F* ^2^)] = 0.041
*wR*(*F* ^2^) = 0.119
*S* = 1.03
11521 reflections
758 parameters
H-atom parameters constrained
Δρ_max_ = 0.31 e Å^−3^
Δρ_min_ = −0.27 e Å^−3^

### 

Data collection: *CrysAlis PRO* (Agilent, 2010 ▶); cell refinement: *CrysAlis PRO*; data reduction: *CrysAlis PRO*; program(s) used to solve structure: *SHELXS97* (Sheldrick, 2008 ▶); program(s) used to refine structure: *SHELXL97* (Sheldrick, 2008 ▶); molecular graphics: *ORTEP-3* (Farrugia, 1997 ▶), *DIAMOND* (Brandenburg, 2006 ▶) and *Qmol* (Gans & Shalloway, 2001 ▶); software used to prepare material for publication: *publCIF* (Westrip, 2010 ▶).

## Supplementary Material

Crystal structure: contains datablock(s) global, I. DOI: 10.1107/S1600536811041808/hg5111sup1.cif

Structure factors: contains datablock(s) I. DOI: 10.1107/S1600536811041808/hg5111Isup2.hkl

Supplementary material file. DOI: 10.1107/S1600536811041808/hg5111Isup3.cml

Additional supplementary materials:  crystallographic information; 3D view; checkCIF report

## Figures and Tables

**Table 1:** Hydrogen-bond geometry (Å, °) *Cg*1 is the centroid of the C39—C44 benzene ring.

*D*—H⋯*A*	*D*—H	H⋯*A*	*D*⋯*A*	*D*—H⋯*A*
C2—H2⋯O3^i^	0.95	2.36	3.2947 (15)	169
C17—H17⋯O3^i^	0.95	2.47	3.3768 (16)	159
C19—H19⋯O1^i^	0.95	2.34	3.2777 (15)	168
C28—H28b⋯O8^ii^	0.98	2.43	3.2910 (19)	146
C34—H34⋯O1^i^	0.95	2.40	3.3501 (16)	178
C36—H36⋯O7^iii^	0.95	2.31	3.2484 (15)	168
C45—H45c⋯O4^iv^	0.98	2.51	3.3207 (19)	140
C51—H51⋯O7^iii^	0.95	2.46	3.3568 (16)	157
C53—H53⋯O5^v^	0.95	2.35	3.2856 (15)	169
C68—H68⋯O5^v^	0.95	2.41	3.3601 (16)	177
C62—H62c⋯*Cg*1	0.98	2.87	3.7658 (17)	152

Symmetry codes: (i) 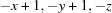; (ii) 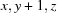; (iii) 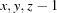; (iv) 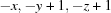; (v) 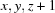.

**Fig. 1.**
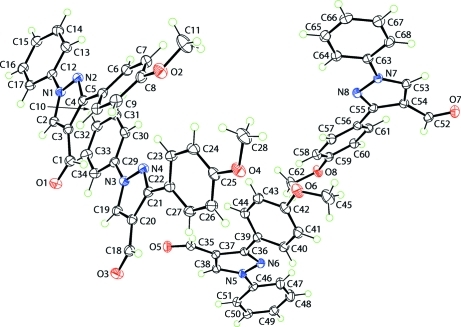
The molecular structures of the four independent molecules comprising the asymmetric unit of (I) showing the atom-labelling scheme and displacement ellipsoids at the 50% probability level.

**Fig. 2.**
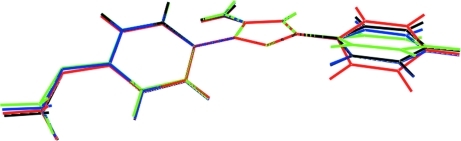
Overlay diagram of the four independent molecules comprising the asymmetric unit of (I). The independent molecule with the O1, O3, O5 (inverted molecule) and O7 atom are illustrated in red, blue, green and black, respectively.

**Fig. 3.**
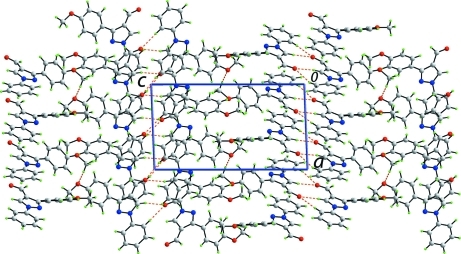
A view of the supramolecular layer in the (0 2 1) plane and sustained by C—H···O interactions (orange dashed lines) in the crystal structure of (I).

**Fig. 4.**
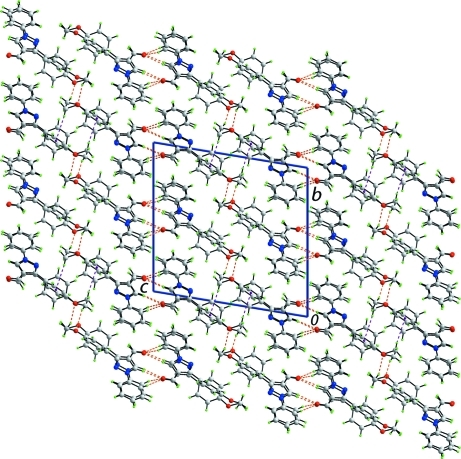
A view in projection down the a axis of the crystal packing in (I) highlighting the stacking of layers. The C—H···O and C—H···π interactions are shown as orange and purple dashed lines, respectively.

## supplementary crystallographic information

### Comment

Pyrazolines and their derivatives have been found to possess a broad spectrum of biological activity such as anti-bacterial, anti-depressant, anti-convulsant, anti-hypertensive, anti-oxidant, and anti-tumour properties (Kaushik *et al.*, 2010; Krishnamurthy *et al.*, 2004). Recent reports shows the potential anti-viral activity of this class of compounds against flavivirus and HIV (Ali *et al.*, 2007). In continuation of structural studies in this area (Prasath *et al.*, 2011), the title compound, (I), was investigated.

Four independent molecules comprise the crystallographic asymmetric unit of (I), Fig. 1. Each molecule comprises a central five-membered pyrazoline ring with a benzene ring attached at the N1-atom and a methoxy-substituted benzene ring at the C3-atom. Differences in the molecules relate primarily to the relative orientations of the various substituents, in particular for the N-bound benzene ring. Thus, the dihedral angles formed between the pyrazoline ring and the N-bound benzene ring are 13.36 (7), 2.54 (7), 15.29 (7) and 1.27 (7)°, respectively, for the independent molecules with the O1, O3, O5 and O7 atoms, respectively. The variation in the dihedral angles formed between the pyrazoline ring and the methoxybenzene ring range from 30.75 (7)° (O1-molecule) to 33.46 (7)° (O7-molecule), *i.e*. display relatively small differences. The dihedral angles formed between the benzene rings within each molecule range from 23.59 (6)° (O1-molecule) to 42.55 (6)° (O5-molecule).

Supramolecular layers in the (0 2 1) plane are formed in the crystal structure *via* C—H···O interactions, Fig. 3 and Table 1. Layers comprise rows of pairs of molecules whereby the aldehyde-O atoms face each other and are connected C—H···O interactions, with the methoxybenzene rings directed to the periphery allowing them to self-associate and thereby propagate the layer. The closest interactions between the layers are of the type C—H···π, Table 1 and Fig 4.

### Experimental

Phosphoryl chloride (11.2 ml) was added drop wise to cold *N*,*N*-dimethylformamide (45 ml) under continuous stirring at 273–278 K for about 30 min. To the reaction mixture, 4-methoxyacetophenone phenylhydrazone (5 g, 33 mmol) was added. The resulting mixture was further stirred at 333 K for 6 h and cooled to room temperature. The crude product was poured into crushed ice which resulted in the deposition of a white precipitate. The resultant solid was filtered, dried and purified by column chromatography using chloroform. Recrystallization was by slow evaporation of a chloroform solution of (I) which yielded colourless prisms. *M*.pt. 403–405 K. Yield: 76%.

### Refinement

Carbon-bound H-atoms were placed in calculated positions [C—H 0.95 to 0.98 Å, *U*_iso_(H) = 1.2 to 1.5*U*_eq_(C)] and were included in the refinement in the riding model approximation.

### Crystal data

**Table:**

C_17_H_14_N_2_O_2_	*Z* = 8
*M**_r_* = 278.30	*F*(000) = 1168
Triclinic, *P*1	*D*_x_ = 1.342 Mg m^−^^3^
Hall symbol: -P 1	Cu *K*α radiation, λ = 1.54184 Å
*a* = 9.9002 (3) Å	Cell parameters from 16969 reflections
*b* = 17.1297 (4) Å	θ = 2.7–76.6°
*c* = 17.1976 (4) Å	µ = 0.72 mm^−^^1^
α = 80.842 (2)°	*T* = 100 K
β = 89.373 (2)°	Prism, colourless
γ = 73.216 (2)°	0.22 × 0.20 × 0.08 mm
*V* = 2754.77 (12) Å^3^	

### Data collection

**Table:**

Agilent SuperNova Dual diffractometer with an Atlas detector	11521 independent reflections
Radiation source: SuperNova (Cu) X-ray Source	10089 reflections with *I* > 2σ(*I*)
Mirror	*R*_int_ = 0.040
Detector resolution: 10.4041 pixels mm^-1^	θ_max_ = 76.8°, θ_min_ = 2.7°
ω scans	*h* = −12→12
Absorption correction: multi-scan (*CrysAlis PRO*; Agilent, 2010)	*k* = −21→21
*T*_min_ = 0.857, *T*_max_ = 0.944	*l* = −21→18
37501 measured reflections	

### Refinement

**Table:**

Refinement on *F*^2^	Secondary atom site location: difference Fourier map
Least-squares matrix: full	Hydrogen site location: inferred from neighbouring sites
*R*[*F*^2^ > 2σ(*F*^2^)] = 0.041	H-atom parameters constrained
*wR*(*F*^2^) = 0.119	*w* = 1/[σ^2^(*F*_o_^2^) + (0.0658*P*)^2^ + 0.494*P*] where *P* = (*F*_o_^2^ + 2*F*_c_^2^)/3
*S* = 1.03	(Δ/σ)_max_ = 0.001
11521 reflections	Δρ_max_ = 0.31 e Å^−^^3^
758 parameters	Δρ_min_ = −0.27 e Å^−^^3^
0 restraints	Extinction correction: *SHELXL97* (Sheldrick, 2008), Fc^*^=kFc[1+0.001xFc^2^λ^3^/sin(2θ)]^-1/4^
Primary atom site location: structure-invariant direct methods	Extinction coefficient: 0.00122 (12)

### Special details

**Table:**

Geometry. All e.s.d.'s (except the e.s.d. in the dihedral angle between two l.s. planes) are estimated using the full covariance matrix. The cell e.s.d.'s are taken into account individually in the estimation of e.s.d.'s in distances, angles and torsion angles; correlations between e.s.d.'s in cell parameters are only used when they are defined by crystal symmetry. An approximate (isotropic) treatment of cell e.s.d.'s is used for estimating e.s.d.'s involving l.s. planes.
Refinement. Refinement of *F*^2^ against ALL reflections. The weighted *R*-factor *wR* and goodness of fit *S* are based on *F*^2^, conventional *R*-factors *R* are based on *F*, with *F* set to zero for negative *F*^2^. The threshold expression of *F*^2^ > σ(*F*^2^) is used only for calculating *R*-factors(gt) *etc*. and is not relevant to the choice of reflections for refinement. *R*-factors based on *F*^2^ are statistically about twice as large as those based on *F*, and *R*- factors based on ALL data will be even larger.

### Fractional atomic coordinates and isotropic or equivalent isotropic displacement parameters (Å^2^)

**Table:**

	*x*	*y*	*z*	*U*_iso_*/*U*_eq_	
O1	0.58482 (9)	0.57924 (6)	0.05987 (5)	0.02499 (19)	
O2	0.64528 (11)	0.68770 (6)	0.48616 (5)	0.0313 (2)	
O3	0.12211 (10)	0.57389 (6)	0.06307 (5)	0.0266 (2)	
O4	0.16811 (11)	0.64833 (6)	0.50406 (5)	0.0295 (2)	
O5	0.31819 (10)	0.08171 (6)	0.06154 (5)	0.02555 (19)	
O6	0.16935 (12)	0.16869 (7)	0.49653 (5)	0.0333 (2)	
O7	0.18467 (10)	−0.07220 (6)	0.94106 (5)	0.02547 (19)	
O8	0.33509 (10)	−0.16770 (6)	0.50911 (5)	0.0258 (2)	
N1	0.98350 (10)	0.43986 (6)	0.16382 (6)	0.0180 (2)	
N2	0.96758 (10)	0.46758 (6)	0.23477 (6)	0.0194 (2)	
N3	0.51269 (10)	0.42232 (6)	0.17081 (6)	0.0186 (2)	
N4	0.49535 (11)	0.44825 (6)	0.24266 (6)	0.0200 (2)	
N5	0.06792 (10)	−0.06535 (6)	0.16798 (6)	0.0183 (2)	
N6	0.05441 (10)	−0.03859 (6)	0.23941 (6)	0.0200 (2)	
N7	0.43830 (10)	0.07212 (6)	0.83291 (6)	0.01756 (19)	
N8	0.44742 (10)	0.04637 (6)	0.76105 (6)	0.0192 (2)	
C1	0.63029 (12)	0.57513 (7)	0.12631 (7)	0.0202 (2)	
H1	0.5681	0.6033	0.1620	0.024*	
C2	0.87069 (12)	0.47580 (7)	0.11461 (7)	0.0184 (2)	
H2	0.8595	0.4661	0.0625	0.022*	
C3	0.77346 (12)	0.52998 (7)	0.15446 (7)	0.0186 (2)	
C4	0.83992 (12)	0.52212 (7)	0.22972 (7)	0.0182 (2)	
C5	0.78876 (12)	0.56447 (7)	0.29713 (7)	0.0192 (2)	
C6	0.83357 (13)	0.52486 (8)	0.37401 (7)	0.0224 (2)	
H6	0.8958	0.4702	0.3820	0.027*	
C7	0.78925 (13)	0.56350 (8)	0.43900 (7)	0.0245 (3)	
H7	0.8208	0.5354	0.4907	0.029*	
C8	0.69837 (13)	0.64360 (8)	0.42762 (7)	0.0233 (2)	
C9	0.65372 (13)	0.68441 (8)	0.35134 (7)	0.0240 (2)	
H9	0.5927	0.7394	0.3434	0.029*	
C10	0.69801 (12)	0.64507 (8)	0.28711 (7)	0.0213 (2)	
H10	0.6662	0.6733	0.2355	0.026*	
C11	0.6982 (2)	0.65301 (11)	0.56458 (8)	0.0427 (4)	
H11A	0.6522	0.6908	0.6006	0.064*	
H11B	0.8004	0.6445	0.5674	0.064*	
H11C	0.6785	0.5999	0.5798	0.064*	
C12	1.11062 (12)	0.37861 (7)	0.15105 (7)	0.0186 (2)	
C13	1.20052 (13)	0.33695 (8)	0.21545 (7)	0.0235 (2)	
H13	1.1771	0.3487	0.2669	0.028*	
C14	1.32521 (14)	0.27783 (8)	0.20339 (8)	0.0259 (3)	
H14	1.3877	0.2493	0.2470	0.031*	
C15	1.35931 (13)	0.26011 (7)	0.12844 (8)	0.0233 (2)	
H15	1.4449	0.2197	0.1207	0.028*	
C16	1.26756 (13)	0.30184 (8)	0.06467 (7)	0.0228 (2)	
H16	1.2902	0.2894	0.0134	0.027*	
C17	1.14274 (13)	0.36175 (8)	0.07553 (7)	0.0210 (2)	
H17	1.0805	0.3907	0.0319	0.025*	
C18	0.17401 (13)	0.57376 (8)	0.12711 (7)	0.0214 (2)	
H18	0.1249	0.6144	0.1572	0.026*	
C19	0.40167 (12)	0.46077 (7)	0.12067 (7)	0.0191 (2)	
H19	0.3909	0.4521	0.0682	0.023*	
C20	0.30584 (12)	0.51559 (7)	0.16038 (7)	0.0191 (2)	
C21	0.37040 (12)	0.50506 (7)	0.23663 (7)	0.0192 (2)	
C22	0.31828 (13)	0.54603 (7)	0.30472 (7)	0.0201 (2)	
C23	0.41356 (13)	0.55878 (8)	0.35722 (7)	0.0229 (2)	
H23	0.5114	0.5436	0.3469	0.028*	
C24	0.36814 (14)	0.59322 (8)	0.42428 (7)	0.0247 (3)	
H24	0.4345	0.6010	0.4596	0.030*	
C25	0.22481 (14)	0.61628 (8)	0.43929 (7)	0.0230 (2)	
C26	0.12803 (13)	0.60534 (8)	0.38659 (7)	0.0245 (2)	
H26	0.0299	0.6225	0.3960	0.029*	
C27	0.17434 (13)	0.56976 (8)	0.32081 (7)	0.0230 (2)	
H27	0.1079	0.5613	0.2861	0.028*	
C28	0.26343 (18)	0.65574 (9)	0.56223 (8)	0.0347 (3)	
H28A	0.2098	0.6798	0.6052	0.052*	
H28B	0.3191	0.6916	0.5383	0.052*	
H28C	0.3268	0.6009	0.5830	0.052*	
C29	0.64008 (12)	0.36156 (7)	0.15769 (7)	0.0188 (2)	
C30	0.74105 (13)	0.32886 (8)	0.21908 (7)	0.0220 (2)	
H30	0.7247	0.3468	0.2688	0.026*	
C31	0.86576 (13)	0.26982 (8)	0.20715 (7)	0.0245 (2)	
H31	0.9347	0.2470	0.2490	0.029*	
C32	0.89020 (13)	0.24395 (8)	0.13456 (8)	0.0245 (3)	
H32	0.9753	0.2033	0.1267	0.029*	
C33	0.78955 (13)	0.27779 (8)	0.07323 (8)	0.0255 (3)	
H33	0.8069	0.2605	0.0233	0.031*	
C34	0.66369 (13)	0.33669 (8)	0.08428 (7)	0.0232 (2)	
H34	0.5950	0.3596	0.0424	0.028*	
C35	0.26001 (12)	0.08421 (7)	0.12434 (7)	0.0205 (2)	
H35	0.2663	0.1258	0.1535	0.025*	
C36	0.14346 (12)	−0.02774 (7)	0.11814 (7)	0.0185 (2)	
H36	0.1663	−0.0373	0.0660	0.022*	
C37	0.18182 (12)	0.02773 (7)	0.15739 (7)	0.0185 (2)	
C38	0.12297 (12)	0.01815 (7)	0.23327 (7)	0.0188 (2)	
C39	0.13082 (12)	0.06021 (7)	0.30083 (7)	0.0196 (2)	
C40	0.02155 (13)	0.07262 (8)	0.35398 (7)	0.0230 (2)	
H40	−0.0597	0.0559	0.3449	0.028*	
C41	0.02964 (14)	0.10902 (8)	0.42009 (7)	0.0261 (3)	
H41	−0.0451	0.1166	0.4558	0.031*	
C42	0.14795 (14)	0.13416 (8)	0.43339 (7)	0.0248 (3)	
C43	0.25728 (14)	0.12274 (8)	0.38051 (7)	0.0246 (3)	
H43	0.3375	0.1405	0.3891	0.030*	
C44	0.24903 (13)	0.08556 (8)	0.31560 (7)	0.0227 (2)	
H44	0.3248	0.0771	0.2805	0.027*	
C45	0.07078 (17)	0.17178 (10)	0.55799 (8)	0.0376 (3)	
H45A	0.0985	0.1978	0.5995	0.056*	
H45B	0.0699	0.1155	0.5800	0.056*	
H45C	−0.0237	0.2041	0.5366	0.056*	
C46	0.00581 (12)	−0.12809 (7)	0.15539 (7)	0.0193 (2)	
C47	−0.03615 (15)	−0.17321 (9)	0.22050 (7)	0.0280 (3)	
H47	−0.0239	−0.1622	0.2720	0.034*	
C48	−0.09642 (16)	−0.23471 (9)	0.20935 (8)	0.0317 (3)	
H48	−0.1259	−0.2657	0.2536	0.038*	
C49	−0.11390 (13)	−0.25129 (8)	0.13443 (8)	0.0250 (3)	
H49	−0.1552	−0.2934	0.1273	0.030*	
C50	−0.07072 (13)	−0.20589 (8)	0.06990 (7)	0.0230 (2)	
H50	−0.0823	−0.2172	0.0185	0.028*	
C51	−0.01042 (13)	−0.14377 (8)	0.07988 (7)	0.0216 (2)	
H51	0.0191	−0.1127	0.0357	0.026*	
C52	0.22576 (12)	−0.06720 (7)	0.87383 (7)	0.0199 (2)	
H52	0.1943	−0.0966	0.8389	0.024*	
C53	0.36385 (12)	0.03428 (7)	0.88344 (7)	0.0181 (2)	
H53	0.3449	0.0424	0.9363	0.022*	
C54	0.31981 (12)	−0.01903 (7)	0.84379 (7)	0.0182 (2)	
C55	0.37520 (12)	−0.00872 (7)	0.76722 (7)	0.0181 (2)	
C56	0.36396 (12)	−0.04936 (7)	0.69937 (7)	0.0185 (2)	
C57	0.35994 (13)	−0.00674 (7)	0.62254 (7)	0.0211 (2)	
H57	0.3627	0.0489	0.6148	0.025*	
C58	0.35193 (13)	−0.04394 (8)	0.55720 (7)	0.0222 (2)	
H58	0.3497	−0.0140	0.5055	0.027*	
C59	0.34727 (12)	−0.12544 (8)	0.56817 (7)	0.0201 (2)	
C60	0.35357 (12)	−0.16930 (7)	0.64453 (7)	0.0200 (2)	
H60	0.3526	−0.2253	0.6521	0.024*	
C61	0.36119 (12)	−0.13156 (7)	0.70916 (7)	0.0194 (2)	
H61	0.3646	−0.1618	0.7608	0.023*	
C62	0.33476 (19)	−0.12768 (10)	0.42996 (8)	0.0379 (3)	
H62A	0.3253	−0.1648	0.3938	0.057*	
H62B	0.4235	−0.1136	0.4209	0.057*	
H62C	0.2553	−0.0771	0.4206	0.057*	
C63	0.50638 (12)	0.13214 (7)	0.84553 (7)	0.0180 (2)	
C64	0.57849 (12)	0.16332 (7)	0.78423 (7)	0.0207 (2)	
H64	0.5823	0.1447	0.7349	0.025*	
C65	0.64514 (13)	0.22220 (8)	0.79583 (7)	0.0233 (2)	
H65	0.6930	0.2446	0.7539	0.028*	
C66	0.64184 (13)	0.24821 (8)	0.86846 (8)	0.0237 (2)	
H66	0.6878	0.2881	0.8764	0.028*	
C67	0.57086 (13)	0.21558 (8)	0.92956 (7)	0.0238 (2)	
H67	0.5692	0.2331	0.9793	0.029*	
C68	0.50222 (13)	0.15758 (8)	0.91878 (7)	0.0224 (2)	
H68	0.4534	0.1357	0.9606	0.027*	

### Atomic displacement parameters (Å^2^)

**Table:**

	*U*^11^	*U*^22^	*U*^33^	*U*^12^	*U*^13^	*U*^23^
O1	0.0239 (4)	0.0262 (5)	0.0230 (4)	−0.0047 (3)	−0.0037 (3)	−0.0027 (3)
O2	0.0369 (5)	0.0334 (5)	0.0229 (4)	−0.0058 (4)	0.0044 (4)	−0.0110 (4)
O3	0.0258 (4)	0.0293 (5)	0.0227 (4)	−0.0042 (4)	−0.0030 (3)	−0.0048 (4)
O4	0.0382 (5)	0.0302 (5)	0.0188 (4)	−0.0047 (4)	−0.0003 (4)	−0.0099 (4)
O5	0.0282 (5)	0.0302 (5)	0.0202 (4)	−0.0123 (4)	0.0055 (3)	−0.0029 (3)
O6	0.0463 (6)	0.0348 (5)	0.0215 (4)	−0.0128 (5)	0.0026 (4)	−0.0112 (4)
O7	0.0294 (5)	0.0277 (5)	0.0212 (4)	−0.0115 (4)	0.0064 (3)	−0.0041 (3)
O8	0.0359 (5)	0.0271 (5)	0.0169 (4)	−0.0115 (4)	0.0029 (3)	−0.0068 (3)
N1	0.0176 (5)	0.0183 (5)	0.0182 (4)	−0.0049 (4)	0.0004 (4)	−0.0038 (4)
N2	0.0193 (5)	0.0206 (5)	0.0191 (5)	−0.0061 (4)	0.0011 (4)	−0.0051 (4)
N3	0.0194 (5)	0.0192 (5)	0.0183 (4)	−0.0064 (4)	0.0009 (4)	−0.0045 (4)
N4	0.0208 (5)	0.0214 (5)	0.0191 (5)	−0.0063 (4)	0.0009 (4)	−0.0064 (4)
N5	0.0181 (5)	0.0193 (5)	0.0172 (4)	−0.0050 (4)	0.0024 (4)	−0.0033 (4)
N6	0.0197 (5)	0.0219 (5)	0.0184 (5)	−0.0052 (4)	0.0031 (4)	−0.0050 (4)
N7	0.0172 (4)	0.0184 (5)	0.0173 (4)	−0.0041 (4)	0.0022 (3)	−0.0057 (4)
N8	0.0195 (5)	0.0209 (5)	0.0177 (4)	−0.0052 (4)	0.0022 (4)	−0.0064 (4)
C1	0.0198 (5)	0.0183 (5)	0.0220 (5)	−0.0053 (4)	0.0006 (4)	−0.0024 (4)
C2	0.0182 (5)	0.0179 (5)	0.0197 (5)	−0.0065 (4)	−0.0001 (4)	−0.0020 (4)
C3	0.0191 (5)	0.0169 (5)	0.0200 (5)	−0.0062 (4)	0.0007 (4)	−0.0015 (4)
C4	0.0179 (5)	0.0174 (5)	0.0206 (5)	−0.0071 (4)	0.0015 (4)	−0.0024 (4)
C5	0.0170 (5)	0.0215 (6)	0.0206 (5)	−0.0077 (4)	0.0022 (4)	−0.0043 (4)
C6	0.0217 (6)	0.0218 (6)	0.0227 (6)	−0.0058 (4)	0.0003 (4)	−0.0019 (5)
C7	0.0265 (6)	0.0279 (6)	0.0198 (5)	−0.0099 (5)	0.0008 (5)	−0.0018 (5)
C8	0.0235 (6)	0.0273 (6)	0.0221 (6)	−0.0103 (5)	0.0048 (5)	−0.0079 (5)
C9	0.0240 (6)	0.0215 (6)	0.0257 (6)	−0.0042 (5)	0.0024 (5)	−0.0055 (5)
C10	0.0211 (6)	0.0219 (6)	0.0208 (5)	−0.0067 (5)	0.0010 (4)	−0.0026 (4)
C11	0.0597 (10)	0.0450 (9)	0.0200 (6)	−0.0070 (8)	0.0033 (6)	−0.0108 (6)
C12	0.0162 (5)	0.0170 (5)	0.0232 (5)	−0.0054 (4)	0.0015 (4)	−0.0039 (4)
C13	0.0228 (6)	0.0248 (6)	0.0211 (6)	−0.0042 (5)	0.0006 (4)	−0.0036 (5)
C14	0.0224 (6)	0.0246 (6)	0.0268 (6)	−0.0024 (5)	−0.0018 (5)	−0.0008 (5)
C15	0.0190 (5)	0.0186 (5)	0.0313 (6)	−0.0036 (4)	0.0026 (5)	−0.0048 (5)
C16	0.0220 (6)	0.0235 (6)	0.0251 (6)	−0.0078 (5)	0.0046 (5)	−0.0080 (5)
C17	0.0195 (5)	0.0226 (6)	0.0216 (5)	−0.0067 (5)	−0.0004 (4)	−0.0040 (4)
C18	0.0208 (6)	0.0212 (6)	0.0217 (5)	−0.0055 (4)	0.0016 (4)	−0.0033 (4)
C19	0.0190 (5)	0.0190 (5)	0.0200 (5)	−0.0064 (4)	−0.0001 (4)	−0.0033 (4)
C20	0.0195 (5)	0.0193 (5)	0.0196 (5)	−0.0071 (4)	0.0014 (4)	−0.0042 (4)
C21	0.0185 (5)	0.0198 (5)	0.0210 (5)	−0.0078 (4)	0.0012 (4)	−0.0044 (4)
C22	0.0221 (6)	0.0201 (5)	0.0194 (5)	−0.0072 (4)	0.0006 (4)	−0.0047 (4)
C23	0.0208 (6)	0.0245 (6)	0.0249 (6)	−0.0083 (5)	−0.0012 (5)	−0.0052 (5)
C24	0.0281 (6)	0.0263 (6)	0.0228 (6)	−0.0117 (5)	−0.0040 (5)	−0.0056 (5)
C25	0.0313 (6)	0.0195 (6)	0.0180 (5)	−0.0064 (5)	0.0004 (5)	−0.0043 (4)
C26	0.0220 (6)	0.0287 (6)	0.0222 (6)	−0.0050 (5)	0.0012 (4)	−0.0068 (5)
C27	0.0214 (6)	0.0276 (6)	0.0211 (5)	−0.0072 (5)	−0.0020 (4)	−0.0065 (5)
C28	0.0543 (9)	0.0325 (7)	0.0212 (6)	−0.0159 (7)	−0.0033 (6)	−0.0090 (5)
C29	0.0176 (5)	0.0166 (5)	0.0225 (5)	−0.0058 (4)	0.0016 (4)	−0.0028 (4)
C30	0.0242 (6)	0.0208 (6)	0.0195 (5)	−0.0056 (5)	0.0006 (4)	−0.0005 (4)
C31	0.0221 (6)	0.0223 (6)	0.0262 (6)	−0.0048 (5)	−0.0022 (5)	0.0020 (5)
C32	0.0191 (6)	0.0197 (6)	0.0328 (6)	−0.0031 (4)	0.0024 (5)	−0.0035 (5)
C33	0.0230 (6)	0.0261 (6)	0.0281 (6)	−0.0053 (5)	0.0023 (5)	−0.0105 (5)
C34	0.0198 (6)	0.0254 (6)	0.0241 (6)	−0.0045 (5)	−0.0015 (4)	−0.0070 (5)
C35	0.0211 (6)	0.0205 (6)	0.0197 (5)	−0.0057 (4)	0.0003 (4)	−0.0028 (4)
C36	0.0176 (5)	0.0183 (5)	0.0183 (5)	−0.0037 (4)	0.0017 (4)	−0.0021 (4)
C37	0.0155 (5)	0.0196 (5)	0.0188 (5)	−0.0027 (4)	0.0014 (4)	−0.0030 (4)
C38	0.0156 (5)	0.0194 (5)	0.0191 (5)	−0.0022 (4)	0.0010 (4)	−0.0022 (4)
C39	0.0195 (5)	0.0186 (5)	0.0190 (5)	−0.0026 (4)	0.0017 (4)	−0.0032 (4)
C40	0.0193 (6)	0.0254 (6)	0.0230 (6)	−0.0038 (5)	0.0026 (4)	−0.0053 (5)
C41	0.0239 (6)	0.0297 (6)	0.0217 (6)	−0.0021 (5)	0.0051 (5)	−0.0063 (5)
C42	0.0316 (7)	0.0222 (6)	0.0189 (5)	−0.0045 (5)	0.0000 (5)	−0.0049 (5)
C43	0.0265 (6)	0.0265 (6)	0.0226 (6)	−0.0103 (5)	0.0006 (5)	−0.0045 (5)
C44	0.0227 (6)	0.0249 (6)	0.0200 (5)	−0.0067 (5)	0.0024 (4)	−0.0028 (5)
C45	0.0476 (9)	0.0374 (8)	0.0220 (6)	−0.0002 (6)	0.0037 (6)	−0.0112 (6)
C46	0.0158 (5)	0.0192 (5)	0.0227 (6)	−0.0045 (4)	0.0017 (4)	−0.0037 (4)
C47	0.0351 (7)	0.0337 (7)	0.0205 (6)	−0.0185 (6)	0.0040 (5)	−0.0040 (5)
C48	0.0400 (8)	0.0343 (7)	0.0261 (6)	−0.0214 (6)	0.0043 (5)	−0.0010 (5)
C49	0.0229 (6)	0.0231 (6)	0.0303 (6)	−0.0087 (5)	0.0017 (5)	−0.0046 (5)
C50	0.0216 (6)	0.0241 (6)	0.0243 (6)	−0.0061 (5)	0.0017 (4)	−0.0074 (5)
C51	0.0212 (6)	0.0226 (6)	0.0213 (6)	−0.0071 (5)	0.0036 (4)	−0.0038 (4)
C52	0.0189 (5)	0.0195 (5)	0.0210 (5)	−0.0045 (4)	0.0016 (4)	−0.0045 (4)
C53	0.0171 (5)	0.0178 (5)	0.0187 (5)	−0.0033 (4)	0.0022 (4)	−0.0038 (4)
C54	0.0166 (5)	0.0183 (5)	0.0189 (5)	−0.0032 (4)	0.0010 (4)	−0.0045 (4)
C55	0.0151 (5)	0.0180 (5)	0.0198 (5)	−0.0026 (4)	0.0006 (4)	−0.0037 (4)
C56	0.0151 (5)	0.0207 (5)	0.0191 (5)	−0.0034 (4)	0.0011 (4)	−0.0049 (4)
C57	0.0228 (6)	0.0194 (5)	0.0218 (5)	−0.0073 (4)	0.0016 (4)	−0.0033 (4)
C58	0.0251 (6)	0.0233 (6)	0.0176 (5)	−0.0070 (5)	0.0012 (4)	−0.0015 (4)
C59	0.0182 (5)	0.0231 (6)	0.0194 (5)	−0.0052 (4)	0.0020 (4)	−0.0062 (4)
C60	0.0211 (5)	0.0192 (5)	0.0201 (5)	−0.0058 (4)	0.0014 (4)	−0.0042 (4)
C61	0.0179 (5)	0.0207 (6)	0.0177 (5)	−0.0027 (4)	0.0007 (4)	−0.0027 (4)
C62	0.0611 (10)	0.0443 (9)	0.0163 (6)	−0.0265 (8)	0.0063 (6)	−0.0079 (6)
C63	0.0154 (5)	0.0161 (5)	0.0214 (5)	−0.0028 (4)	0.0002 (4)	−0.0038 (4)
C64	0.0205 (5)	0.0188 (5)	0.0208 (5)	−0.0031 (4)	0.0008 (4)	−0.0022 (4)
C65	0.0207 (6)	0.0204 (6)	0.0267 (6)	−0.0050 (5)	0.0015 (5)	0.0003 (5)
C66	0.0195 (6)	0.0190 (6)	0.0327 (6)	−0.0053 (4)	−0.0015 (5)	−0.0050 (5)
C67	0.0235 (6)	0.0238 (6)	0.0258 (6)	−0.0066 (5)	0.0014 (5)	−0.0095 (5)
C68	0.0213 (6)	0.0246 (6)	0.0227 (6)	−0.0074 (5)	0.0042 (4)	−0.0071 (5)

### Geometric parameters (Å, °)

**Table:**

O1—C1	1.2168 (15)	C28—H28A	0.9800
O2—C8	1.3645 (15)	C28—H28B	0.9800
O2—C11	1.4238 (17)	C28—H28C	0.9800
O3—C18	1.2198 (15)	C29—C30	1.3926 (17)
O4—C25	1.3640 (15)	C29—C34	1.3919 (16)
O4—C28	1.4274 (16)	C30—C31	1.3888 (17)
O5—C35	1.2193 (15)	C30—H30	0.9500
O6—C42	1.3623 (15)	C31—C32	1.3859 (18)
O6—C45	1.4274 (17)	C31—H31	0.9500
O7—C52	1.2199 (15)	C32—C33	1.3915 (18)
O8—C59	1.3627 (14)	C32—H32	0.9500
O8—C62	1.4229 (15)	C33—C34	1.3916 (17)
N1—C2	1.3418 (15)	C33—H33	0.9500
N1—N2	1.3698 (13)	C34—H34	0.9500
N1—C12	1.4274 (14)	C35—C37	1.4513 (16)
N2—C4	1.3314 (15)	C35—H35	0.9500
N3—C19	1.3443 (15)	C36—C37	1.3859 (16)
N3—N4	1.3711 (13)	C36—H36	0.9500
N3—C29	1.4266 (15)	C37—C38	1.4282 (15)
N4—C21	1.3280 (16)	C38—C39	1.4751 (16)
N5—C36	1.3407 (15)	C39—C40	1.3993 (16)
N5—N6	1.3701 (13)	C39—C44	1.3993 (17)
N5—C46	1.4278 (15)	C40—C41	1.3943 (17)
N6—C38	1.3278 (16)	C40—H40	0.9500
N7—C53	1.3421 (15)	C41—C42	1.3923 (19)
N7—N8	1.3703 (13)	C41—H41	0.9500
N7—C63	1.4259 (15)	C42—C43	1.3963 (18)
N8—C55	1.3302 (15)	C43—C44	1.3843 (17)
C1—C3	1.4506 (16)	C43—H43	0.9500
C1—H1	0.9500	C44—H44	0.9500
C2—C3	1.3891 (16)	C45—H45A	0.9800
C2—H2	0.9500	C45—H45B	0.9800
C3—C4	1.4277 (16)	C45—H45C	0.9800
C4—C5	1.4728 (15)	C46—C51	1.3875 (16)
C5—C10	1.3980 (17)	C46—C47	1.3871 (17)
C5—C6	1.3995 (16)	C47—C48	1.3900 (18)
C6—C7	1.3916 (17)	C47—H47	0.9500
C6—H6	0.9500	C48—C49	1.3858 (18)
C7—C8	1.3914 (18)	C48—H48	0.9500
C7—H7	0.9500	C49—C50	1.3879 (18)
C8—C9	1.3954 (18)	C49—H49	0.9500
C9—C10	1.3864 (16)	C50—C51	1.3946 (17)
C9—H9	0.9500	C50—H50	0.9500
C10—H10	0.9500	C51—H51	0.9500
C11—H11A	0.9800	C52—C54	1.4532 (16)
C11—H11B	0.9800	C52—H52	0.9500
C11—H11C	0.9800	C53—C54	1.3869 (16)
C12—C13	1.3897 (17)	C53—H53	0.9500
C12—C17	1.3887 (16)	C54—C55	1.4256 (16)
C13—C14	1.3899 (17)	C55—C56	1.4712 (15)
C13—H13	0.9500	C56—C61	1.3994 (16)
C14—C15	1.3869 (18)	C56—C57	1.3993 (16)
C14—H14	0.9500	C57—C58	1.3907 (16)
C15—C16	1.3914 (18)	C57—H57	0.9500
C15—H15	0.9500	C58—C59	1.3928 (17)
C16—C17	1.3924 (17)	C58—H58	0.9500
C16—H16	0.9500	C59—C60	1.3977 (16)
C17—H17	0.9500	C60—C61	1.3847 (15)
C18—C20	1.4517 (17)	C60—H60	0.9500
C18—H18	0.9500	C61—H61	0.9500
C19—C20	1.3870 (16)	C62—H62A	0.9800
C19—H19	0.9500	C62—H62B	0.9800
C20—C21	1.4274 (16)	C62—H62C	0.9800
C21—C22	1.4728 (16)	C63—C64	1.3895 (16)
C22—C23	1.3974 (16)	C63—C68	1.3934 (16)
C22—C27	1.4019 (17)	C64—C65	1.3942 (17)
C23—C24	1.3901 (17)	C64—H64	0.9500
C23—H23	0.9500	C65—C66	1.3884 (17)
C24—C25	1.3917 (19)	C65—H65	0.9500
C24—H24	0.9500	C66—C67	1.3907 (18)
C25—C26	1.3969 (17)	C66—H66	0.9500
C26—C27	1.3814 (17)	C67—C68	1.3908 (17)
C26—H26	0.9500	C67—H67	0.9500
C27—H27	0.9500	C68—H68	0.9500
			
C8—O2—C11	117.69 (11)	C30—C31—H31	119.8
C25—O4—C28	117.49 (11)	C31—C32—C33	119.74 (11)
C42—O6—C45	117.85 (12)	C31—C32—H32	120.1
C59—O8—C62	118.04 (10)	C33—C32—H32	120.1
C2—N1—N2	112.50 (9)	C32—C33—C34	120.67 (12)
C2—N1—C12	128.52 (10)	C32—C33—H33	119.7
N2—N1—C12	118.98 (9)	C34—C33—H33	119.7
C4—N2—N1	105.23 (9)	C29—C34—C33	118.94 (11)
C19—N3—N4	112.40 (10)	C29—C34—H34	120.5
C19—N3—C29	128.62 (10)	C33—C34—H34	120.5
N4—N3—C29	118.97 (9)	O5—C35—C37	124.19 (11)
C21—N4—N3	105.19 (9)	O5—C35—H35	117.9
C36—N5—N6	112.70 (9)	C37—C35—H35	117.9
C36—N5—C46	128.23 (10)	N5—C36—C37	106.73 (10)
N6—N5—C46	119.06 (9)	N5—C36—H36	126.6
C38—N6—N5	104.93 (9)	C37—C36—H36	126.6
C53—N7—N8	112.39 (9)	C36—C37—C38	104.83 (10)
C53—N7—C63	128.65 (10)	C36—C37—C35	125.55 (11)
N8—N7—C63	118.96 (9)	C38—C37—C35	129.58 (11)
C55—N8—N7	105.10 (9)	N6—C38—C37	110.80 (10)
O1—C1—C3	124.13 (11)	N6—C38—C39	119.66 (10)
O1—C1—H1	117.9	C37—C38—C39	129.53 (11)
C3—C1—H1	117.9	C40—C39—C44	118.19 (11)
N1—C2—C3	106.80 (10)	C40—C39—C38	120.32 (11)
N1—C2—H2	126.6	C44—C39—C38	121.44 (10)
C3—C2—H2	126.6	C41—C40—C39	121.24 (12)
C2—C3—C4	104.91 (10)	C41—C40—H40	119.4
C2—C3—C1	125.25 (11)	C39—C40—H40	119.4
C4—C3—C1	129.42 (11)	C42—C41—C40	119.59 (11)
N2—C4—C3	110.55 (10)	C42—C41—H41	120.2
N2—C4—C5	119.40 (10)	C40—C41—H41	120.2
C3—C4—C5	130.05 (11)	O6—C42—C41	125.01 (12)
C10—C5—C6	117.88 (11)	O6—C42—C43	115.20 (12)
C10—C5—C4	122.00 (11)	C41—C42—C43	119.78 (11)
C6—C5—C4	120.10 (11)	C44—C43—C42	120.18 (12)
C7—C6—C5	121.66 (12)	C44—C43—H43	119.9
C7—C6—H6	119.2	C42—C43—H43	119.9
C5—C6—H6	119.2	C43—C44—C39	121.02 (11)
C8—C7—C6	119.44 (11)	C43—C44—H44	119.5
C8—C7—H7	120.3	C39—C44—H44	119.5
C6—C7—H7	120.3	O6—C45—H45A	109.5
O2—C8—C7	125.24 (12)	O6—C45—H45B	109.5
O2—C8—C9	115.04 (12)	H45A—C45—H45B	109.5
C7—C8—C9	119.72 (11)	O6—C45—H45C	109.5
C10—C9—C8	120.28 (12)	H45A—C45—H45C	109.5
C10—C9—H9	119.9	H45B—C45—H45C	109.5
C8—C9—H9	119.9	C51—C46—C47	121.16 (11)
C9—C10—C5	121.01 (11)	C51—C46—N5	120.56 (11)
C9—C10—H10	119.5	C47—C46—N5	118.28 (11)
C5—C10—H10	119.5	C46—C47—C48	119.06 (12)
O2—C11—H11A	109.5	C46—C47—H47	120.5
O2—C11—H11B	109.5	C48—C47—H47	120.5
H11A—C11—H11B	109.5	C49—C48—C47	120.74 (12)
O2—C11—H11C	109.5	C49—C48—H48	119.6
H11A—C11—H11C	109.5	C47—C48—H48	119.6
H11B—C11—H11C	109.5	C48—C49—C50	119.52 (12)
C13—C12—C17	121.26 (11)	C48—C49—H49	120.2
C13—C12—N1	118.60 (10)	C50—C49—H49	120.2
C17—C12—N1	120.14 (10)	C49—C50—C51	120.58 (11)
C12—C13—C14	118.95 (11)	C49—C50—H50	119.7
C12—C13—H13	120.5	C51—C50—H50	119.7
C14—C13—H13	120.5	C46—C51—C50	118.94 (11)
C15—C14—C13	120.70 (12)	C46—C51—H51	120.5
C15—C14—H14	119.6	C50—C51—H51	120.5
C13—C14—H14	119.6	O7—C52—C54	124.09 (11)
C14—C15—C16	119.64 (11)	O7—C52—H52	118.0
C14—C15—H15	120.2	C54—C52—H52	118.0
C16—C15—H15	120.2	N7—C53—C54	106.93 (10)
C15—C16—C17	120.46 (11)	N7—C53—H53	126.5
C15—C16—H16	119.8	C54—C53—H53	126.5
C17—C16—H16	119.8	C53—C54—C55	104.80 (10)
C12—C17—C16	118.98 (11)	C53—C54—C52	125.68 (10)
C12—C17—H17	120.5	C55—C54—C52	129.25 (11)
C16—C17—H17	120.5	N8—C55—C54	110.78 (10)
O3—C18—C20	124.32 (11)	N8—C55—C56	119.36 (10)
O3—C18—H18	117.8	C54—C55—C56	129.87 (11)
C20—C18—H18	117.8	C61—C56—C57	118.11 (10)
N3—C19—C20	106.76 (10)	C61—C56—C55	121.66 (10)
N3—C19—H19	126.6	C57—C56—C55	120.20 (11)
C20—C19—H19	126.6	C58—C57—C56	121.55 (11)
C19—C20—C21	104.93 (10)	C58—C57—H57	119.2
C19—C20—C18	125.71 (11)	C56—C57—H57	119.2
C21—C20—C18	129.24 (11)	C57—C58—C59	119.47 (11)
N4—C21—C20	110.72 (10)	C57—C58—H58	120.3
N4—C21—C22	119.35 (10)	C59—C58—H58	120.3
C20—C21—C22	129.93 (11)	O8—C59—C58	124.94 (11)
C23—C22—C27	118.28 (11)	O8—C59—C60	115.40 (11)
C23—C22—C21	120.06 (11)	C58—C59—C60	119.66 (11)
C27—C22—C21	121.60 (11)	C61—C60—C59	120.35 (11)
C24—C23—C22	121.35 (11)	C61—C60—H60	119.8
C24—C23—H23	119.3	C59—C60—H60	119.8
C22—C23—H23	119.3	C60—C61—C56	120.83 (11)
C23—C24—C25	119.53 (11)	C60—C61—H61	119.6
C23—C24—H24	120.2	C56—C61—H61	119.6
C25—C24—H24	120.2	O8—C62—H62A	109.5
O4—C25—C24	124.89 (11)	O8—C62—H62B	109.5
O4—C25—C26	115.31 (11)	H62A—C62—H62B	109.5
C24—C25—C26	119.79 (11)	O8—C62—H62C	109.5
C27—C26—C25	120.28 (12)	H62A—C62—H62C	109.5
C27—C26—H26	119.9	H62B—C62—H62C	109.5
C25—C26—H26	119.9	C64—C63—C68	120.99 (11)
C26—C27—C22	120.76 (11)	C64—C63—N7	118.89 (10)
C26—C27—H27	119.6	C68—C63—N7	120.11 (10)
C22—C27—H27	119.6	C63—C64—C65	119.39 (11)
O4—C28—H28A	109.5	C63—C64—H64	120.3
O4—C28—H28B	109.5	C65—C64—H64	120.3
H28A—C28—H28B	109.5	C66—C65—C64	120.28 (11)
O4—C28—H28C	109.5	C66—C65—H65	119.9
H28A—C28—H28C	109.5	C64—C65—H65	119.9
H28B—C28—H28C	109.5	C65—C66—C67	119.64 (11)
C30—C29—C34	120.77 (11)	C65—C66—H66	120.2
C30—C29—N3	118.84 (10)	C67—C66—H66	120.2
C34—C29—N3	120.37 (11)	C68—C67—C66	120.89 (11)
C31—C30—C29	119.51 (11)	C68—C67—H67	119.6
C31—C30—H30	120.2	C66—C67—H67	119.6
C29—C30—H30	120.2	C67—C68—C63	118.80 (11)
C32—C31—C30	120.35 (11)	C67—C68—H68	120.6
C32—C31—H31	119.8	C63—C68—H68	120.6
			
C2—N1—N2—C4	0.54 (13)	C31—C32—C33—C34	−0.8 (2)
C12—N1—N2—C4	−178.87 (10)	C30—C29—C34—C33	0.66 (18)
C19—N3—N4—C21	−0.60 (13)	N3—C29—C34—C33	179.48 (11)
C29—N3—N4—C21	178.73 (10)	C32—C33—C34—C29	0.23 (19)
C36—N5—N6—C38	0.57 (13)	N6—N5—C36—C37	−0.60 (13)
C46—N5—N6—C38	179.47 (10)	C46—N5—C36—C37	−179.37 (11)
C53—N7—N8—C55	0.51 (13)	N5—C36—C37—C38	0.36 (13)
C63—N7—N8—C55	179.86 (10)	N5—C36—C37—C35	−177.40 (11)
N2—N1—C2—C3	−0.45 (13)	O5—C35—C37—C36	−10.59 (19)
C12—N1—C2—C3	178.89 (10)	O5—C35—C37—C38	172.22 (12)
N1—C2—C3—C4	0.17 (12)	N5—N6—C38—C37	−0.32 (13)
N1—C2—C3—C1	−172.96 (11)	N5—N6—C38—C39	−179.55 (10)
O1—C1—C3—C2	−10.26 (19)	C36—C37—C38—N6	−0.02 (13)
O1—C1—C3—C4	178.35 (12)	C35—C37—C38—N6	177.61 (11)
N1—N2—C4—C3	−0.42 (12)	C36—C37—C38—C39	179.12 (11)
N1—N2—C4—C5	−179.88 (10)	C35—C37—C38—C39	−3.2 (2)
C2—C3—C4—N2	0.16 (13)	N6—C38—C39—C40	−30.22 (17)
C1—C3—C4—N2	172.90 (11)	C37—C38—C39—C40	150.71 (12)
C2—C3—C4—C5	179.55 (11)	N6—C38—C39—C44	147.06 (12)
C1—C3—C4—C5	−7.7 (2)	C37—C38—C39—C44	−32.01 (19)
N2—C4—C5—C10	148.19 (11)	C44—C39—C40—C41	−0.16 (18)
C3—C4—C5—C10	−31.15 (18)	C38—C39—C40—C41	177.20 (11)
N2—C4—C5—C6	−30.21 (16)	C39—C40—C41—C42	0.50 (19)
C3—C4—C5—C6	150.45 (12)	C45—O6—C42—C41	6.82 (19)
C10—C5—C6—C7	0.46 (18)	C45—O6—C42—C43	−171.74 (12)
C4—C5—C6—C7	178.92 (11)	C40—C41—C42—O6	−178.49 (12)
C5—C6—C7—C8	−0.16 (19)	C40—C41—C42—C43	0.01 (19)
C11—O2—C8—C7	7.2 (2)	O6—C42—C43—C44	177.80 (12)
C11—O2—C8—C9	−173.54 (13)	C41—C42—C43—C44	−0.84 (19)
C6—C7—C8—O2	178.76 (12)	C42—C43—C44—C39	1.20 (19)
C6—C7—C8—C9	−0.50 (19)	C40—C39—C44—C43	−0.69 (18)
O2—C8—C9—C10	−178.47 (11)	C38—C39—C44—C43	−178.02 (11)
C7—C8—C9—C10	0.87 (19)	C36—N5—C46—C51	−15.70 (18)
C8—C9—C10—C5	−0.57 (19)	N6—N5—C46—C51	165.59 (10)
C6—C5—C10—C9	−0.09 (17)	C36—N5—C46—C47	163.62 (12)
C4—C5—C10—C9	−178.52 (11)	N6—N5—C46—C47	−15.09 (16)
C2—N1—C12—C13	−166.13 (12)	C51—C46—C47—C48	−0.5 (2)
N2—N1—C12—C13	13.17 (16)	N5—C46—C47—C48	−179.80 (12)
C2—N1—C12—C17	14.04 (18)	C46—C47—C48—C49	0.3 (2)
N2—N1—C12—C17	−166.66 (10)	C47—C48—C49—C50	0.0 (2)
C17—C12—C13—C14	0.48 (19)	C48—C49—C50—C51	−0.2 (2)
N1—C12—C13—C14	−179.34 (11)	C47—C46—C51—C50	0.30 (19)
C12—C13—C14—C15	−0.43 (19)	N5—C46—C51—C50	179.60 (11)
C13—C14—C15—C16	−0.1 (2)	C49—C50—C51—C46	0.04 (19)
C14—C15—C16—C17	0.68 (19)	N8—N7—C53—C54	−0.43 (13)
C13—C12—C17—C16	0.04 (18)	C63—N7—C53—C54	−179.69 (11)
N1—C12—C17—C16	179.87 (10)	N7—C53—C54—C55	0.17 (12)
C15—C16—C17—C12	−0.63 (18)	N7—C53—C54—C52	−174.29 (11)
N4—N3—C19—C20	0.55 (13)	O7—C52—C54—C53	−7.23 (19)
C29—N3—C19—C20	−178.71 (11)	O7—C52—C54—C55	179.70 (12)
N3—C19—C20—C21	−0.26 (13)	N7—N8—C55—C54	−0.39 (12)
N3—C19—C20—C18	176.08 (11)	N7—N8—C55—C56	−179.85 (9)
O3—C18—C20—C19	10.4 (2)	C53—C54—C55—N8	0.15 (13)
O3—C18—C20—C21	−174.19 (12)	C52—C54—C55—N8	174.33 (11)
N3—N4—C21—C20	0.41 (13)	C53—C54—C55—C56	179.53 (11)
N3—N4—C21—C22	179.84 (10)	C52—C54—C55—C56	−6.3 (2)
C19—C20—C21—N4	−0.10 (13)	N8—C55—C56—C61	145.28 (11)
C18—C20—C21—N4	−176.27 (12)	C54—C55—C56—C61	−34.07 (18)
C19—C20—C21—C22	−179.45 (11)	N8—C55—C56—C57	−32.82 (16)
C18—C20—C21—C22	4.4 (2)	C54—C55—C56—C57	147.84 (12)
N4—C21—C22—C23	31.51 (17)	C61—C56—C57—C58	0.66 (18)
C20—C21—C22—C23	−149.18 (13)	C55—C56—C57—C58	178.82 (11)
N4—C21—C22—C27	−145.56 (12)	C56—C57—C58—C59	0.24 (18)
C20—C21—C22—C27	33.74 (19)	C62—O8—C59—C58	3.63 (19)
C27—C22—C23—C24	0.68 (19)	C62—O8—C59—C60	−177.14 (12)
C21—C22—C23—C24	−176.49 (11)	C57—C58—C59—O8	177.91 (11)
C22—C23—C24—C25	−0.60 (19)	C57—C58—C59—C60	−1.29 (18)
C28—O4—C25—C24	−2.59 (19)	O8—C59—C60—C61	−177.81 (10)
C28—O4—C25—C26	176.06 (11)	C58—C59—C60—C61	1.46 (18)
C23—C24—C25—O4	177.99 (12)	C59—C60—C61—C56	−0.56 (18)
C23—C24—C25—C26	−0.60 (19)	C57—C56—C61—C60	−0.49 (17)
O4—C25—C26—C27	−177.00 (12)	C55—C56—C61—C60	−178.63 (10)
C24—C25—C26—C27	1.72 (19)	C53—N7—C63—C64	179.95 (11)
C25—C26—C27—C22	−1.7 (2)	N8—N7—C63—C64	0.72 (15)
C23—C22—C27—C26	0.45 (19)	C53—N7—C63—C68	1.10 (18)
C21—C22—C27—C26	177.58 (12)	N8—N7—C63—C68	−178.12 (10)
C19—N3—C29—C30	−179.02 (11)	C68—C63—C64—C65	−1.34 (17)
N4—N3—C29—C30	1.77 (15)	N7—C63—C64—C65	179.83 (10)
C19—N3—C29—C34	2.15 (18)	C63—C64—C65—C66	1.27 (18)
N4—N3—C29—C34	−177.06 (11)	C64—C65—C66—C67	−0.40 (18)
C34—C29—C30—C31	−1.00 (18)	C65—C66—C67—C68	−0.44 (19)
N3—C29—C30—C31	−179.83 (11)	C66—C67—C68—C63	0.38 (19)
C29—C30—C31—C32	0.44 (18)	C64—C63—C68—C67	0.51 (18)
C30—C31—C32—C33	0.44 (19)	N7—C63—C68—C67	179.33 (11)

### Hydrogen-bond geometry (Å, °)

**Table:**

Cg1 is the centroid of the C39—C44 benzene ring.

**Table:**

*D*—H···*A*	*D*—H	H···*A*	*D*···*A*	*D*—H···*A*
C2—H2···O3^i^	0.95	2.36	3.2947 (15)	169
C17—H17···O3^i^	0.95	2.47	3.3768 (16)	159
C19—H19···O1^i^	0.95	2.34	3.2777 (15)	168
C28—H28b···O8^ii^	0.98	2.43	3.2910 (19)	146
C34—H34···O1^i^	0.95	2.40	3.3501 (16)	178
C36—H36···O7^iii^	0.95	2.31	3.2484 (15)	168
C45—H45c···O4^iv^	0.98	2.51	3.3207 (19)	140
C51—H51···O7^iii^	0.95	2.46	3.3568 (16)	157
C53—H53···O5^v^	0.95	2.35	3.2856 (15)	169
C68—H68···O5^v^	0.95	2.41	3.3601 (16)	177
C62—H62c···Cg1	0.98	2.87	3.7658 (17)	152

Symmetry codes: (i) −*x*+1, −*y*+1, −*z*; (ii) *x*, *y*+1, *z*; (iii) *x*, *y*, *z*−1; (iv) −*x*, −*y*+1, −*z*+1; (v) *x*, *y*, *z*+1.
